# Isolation, Morphological and Molecular–Phenological Identification of Nematophagous Fungi Inhabiting the Soils of Agricultural Lands in Southern Kazakhstan

**DOI:** 10.3390/jof11010042

**Published:** 2025-01-07

**Authors:** Gulzat Kanalbek, Akniyet Zhanuzak, Dmitry Faleev, Aidos Nusupov, Karlygash Mukhatayeva, Kenzhe-Karim Boguspaev

**Affiliations:** 1 Faculty of Biology and Biotechnology, Al-Fabari Kazakh National University, Almaty 050040, Kazakhstan; 2 Faculty of Technology, Almaty Technological University, Almaty 050012, Kazakhstan

**Keywords:** soil, nematodes, nematophagous fungi (NF), phylogeny, nematicides

## Abstract

The aim of the present research is the isolation and morphological and molecular–phenological identification of nematophagous fungi of Southern Kazakhstan for the production of effective bionematicides on their basis. Nematophagous fungi, which include nematode-trapping, ovicidal, endoparasitic, toxin-producing, and special substance-producing fungi, are among the most effective biological agents in controlling phytoparasitic nematodes. To isolate and characterize nematophagous fungi, soil samples were collected at 12 sites in three regions of Southern Kazakhstan. The samples were collected using the envelope method. The content of nematophagous fungi in the samples was determined using the standard surface sowing technique. The obtained strains of nematophagous fungi were identified. The attractive and nematophagous activity of the obtained fungal strains was determined by using standard methods. In experiments on the isolation and morphological identification of nematophagous fungi, the nematode species *Meloidogyne incognita* was used. Identification of the strains was carried out by the method of determining the direct nucleotide sequence of the region of the nuclear ribosomal internal transcribed spacer, followed by determination of nucleotide identity with sequences deposited in the international GeneBank database. As a result, the following species of nematophagous fungi living in the soils of agricultural lands in Southern Kazakhstan were identified: *Orbilia oligospora*, *Duddingtonia flagrans*, *Orbilia oligospora*, and *Arthrobotrys superba*.

## 1. Introduction

Large areas of Southern Kazakhstan are used for the cultivation of agricultural crops, including cereals (wheat, barley, and corn), vegetables (tomatoes, cucumbers, potatoes, and cabbages), legumes (soybeans), as well as onions, watermelons, etc.

No specific statistics regarding the damage caused by nematodes on individual farms exist. However, it is known that the plant pathogenic golden potato nematode, *Globodera rostochiensis* Woll., is a quarantine organism in Kazakhstan. This nematode causes potato globoderosis disease and is highly detrimental to infested plants. In Kazakhstan, potatoes are grown mainly on private farms (70%) in monoculture with limited plant protection products. Globoderosis, caused by *Globodera rostochiensis*, can affect up to 40% of the crop and has registered outbreaks in Almaty and East Kazakhstan regions [1]. Beet cyst-forming nematode *Heterodera schachtii* Schmidt was brought to South Kazakhstan from Ukraine in 1960–1970, which of course had a detrimental effect on the yield of sugar beet [2]. Analysis of grain-producing areas of Northern Kazakhstan revealed that 90% of soil samples were infested by the phytoparasitic nematodes of thirteen genera, including Pratylenchus, Heterodera, Geocenamus, Ditylenchus, Helicotylenchus, Rotylenchus, Pratylenchoides, and Tylenchorhynchus, which were detected and identified based on morphological and molecular analysis [3].

There are over 3000 known species of phytoparasitic nematodes that affect nearly all cultivated plants. At the turn of the 20th to 21st century, annual global losses of agricultural products were estimated at 10–14%, equivalent to 100–125 billion USD. By the beginning of the 2020s, this figure had risen to 1500 billion USD [4,5,6]. Since the 1950s–70s, nematodes were primarily controlled using chemical nematicides [7]. However, over time, shortcomings of these chemical preparations became apparent, including their toxicity to humans and animals, reduced effectiveness due to the development of nematicide-resistant nematodes, soil pollution from toxic substances, and negative impacts on ecosystems [8,9]. Currently, biological methods are proposed to solve these problems: (1) breeding varieties of agricultural crops resistant to nematodes [10]; (2) use of embryophytes as baits and antagonists of nematodes; (3) development of methods for using nematophagous fungi (NF) [11].

Among these proposed methods, NF is considered the most promising for nematode biocontrol. They can be used to create new biological control agents (bionematicides) capable of effectively combating nematode infections in both closed and open ground [12,13]. In this context, isolating local strains of NF that are well adapted to the conditions of Southern Kazakhstan is essential for ensuring the effectiveness of bionematicides.

NF are widespread microorganisms that naturally act as antagonists of nematodes, feeding on various life stages of these organisms [14,15]. Many of these fungi are facultative parasites, capable of transitioning from a saprophytic to a parasitic phase when in contact with their hosts. During this transition, they develop specialized infection structures, which are used to classify them based on their predatory strategies [16]. Nematode-trapping fungi utilize modified hyphae such as adhesive networks, adhesive knobs, or constricting rings to capture, immobilize, and digest nematodes using enzymes like proteases, chitinases, and lipases. Toxin-producing fungi attack nematodes by secreting toxins that immobilize their prey, enabling hyphal penetration through the cuticle [17]. Fungi with specialized attack devices damage the nematode cuticle, facilitating colonization and consumption [18]. Ovicidal fungi use similar mechanisms to prey on nematode eggs, cysts, and females. Nematophagous fungi are commonly used in the biocontrol of phytonematodes, helping protect plants from nematode parasites. They are often found in the rhizosphere of crop plants and can colonize plant roots [19,20]. Since plant-parasitic nematodes primarily target roots, the ability of these fungi to colonize root systems is particularly appealing for agricultural applications [19]. Another beneficial trait is the ability to produce many chlamydospores in both soil and culture. The outer shells of these chlamydospores provide reliable protection against pests (e.g., mites and amoebas) and other soil fauna. Moreover, chlamydospores can withstand prolonged drought and other unfavorable environmental conditions [21]. Most studies exploring the relationship between phytoparasitic nematodes and NF aim to find solutions for developing effective and sustainable bionematicides [22,23,24]. For example, a group of scientists from the United States studied the molecular basis of predator-prey coevolution using nematodes of *Caenorhabditis elegans* Dougherty and their response to the NF *Arthrobotrys oligospora* Fresen. This research showed that C. elegans nematodes were attracted to volatile compounds produced by *A. oligospora*. Gas chromatography-mass spectrometry analysis of volatile metabolites from *A. oligospora* identified an odorant that mimics food cues attractive to nematodes [25].

Chinese scientists conducted proteomic and quantitative polymerase chain reaction (qPCR) analyses of *A. oligospora* during the formation of trapping loops for nematodes. They found that nematode extracts significantly upregulated 90 genes in the early stages of trap formation, with most of these genes involved in translation, amino acid metabolism, carbohydrate metabolism, and cell wall and membrane biogenesis. This study will facilitate the identification of pathogenicity-related genes and provide a broader understanding of the molecular and evolutionary mechanisms underlying fungal-nematode interactions [26]. Previous experiments demonstrated the nematophagous activity of a Kyrgyz *A. oligospora* isolate, which was able to trap up to 85.7% of garlic nematodes in the rhizosphere soil of potted potato plants, making it a potentially promising resource for future bionematicide development [27].

The present research aims to isolate, morphologically, and molecularly characterize the NF of Southern Kazakhstan, ultimately leading to the development of an effective bionematicide based on these findings. Recommendations will be developed for the use of each isolated strain of predatory fungi from a specific region of Southern Kazakhstan for the control of phytoparasitic nematodes living in these soils.

This study represents the first exploration of nematophagous fungi inhabiting the soils of Kazakhstan.

## 2. Materials and Methods

### 2.1. Soil Sampling for the Isolation of NF

**Figure 1:**
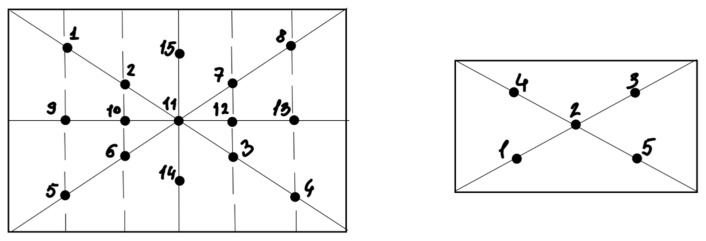
Envelope sampling method used in the research.

To isolate and characterize NF, soil samples were collected from 12 sites across three regions of Southern Kazakhstan: Kyzylorda, Almaty, and Zhambyl (Table 1). All samples were obtained from areas where various crops are cultivated. The samples were collected using the envelope method (Figure 1) with an earth auger at 10–15 cm depth. Sterile tools and containers were used to ensure contamination-free collection. Each sample, weighing up to two kg, was placed in sterile 50 mL test tubes with stoppers (BIOLOGIX^®^, Jinan, Shandong, (China) to prevent drying. The samples were then labeled and stored in a refrigerator at a temperature of 4–5 °C for no more than 7 days.

Characteristics of the soil samples and the locations from which they were collected:

Sample A-4 was taken from a private garden in Kyzylorda. The soil has a light straw color and a high content of carbonates, with an alkaline reaction in the soil solution. It contains water-soluble salts and has a layered composition, along with a low humus content. Additionally, the soil has a loose texture and has been frequently fertilized to enhance crop yields.

Sample B-3 was collected from a potato field in the village of Orazaly in the Zhambyl region; this soil sample exhibits a brownish-gray color and a loose texture. It is loamy, with a humus content of at least 2% and a pH level ranging from 4.5 to 6.3.

Sample C-1 was collected in a potato field at the Kazakh Research Institute of Potato and Vegetable Growing in the Almaty region. The soil is classified as medium and light loamy, with a humus content between 2.5% and 3.0% and a pH of at least 5.6. The color is grayish, and the density is loose.

Sample C-10 was collected from a site designated for corn cultivation in the Kazakh Research Institute of Potato and Vegetable Growing in the Almaty region. The soil characteristics of this plot are identical to those of the C-1 sample collection site.

### 2.2. Isolation of Predatory Fungi

Water agar (WA) was used to isolate NF. To prepare the WA, 20 g of agar-agar was dissolved in 1 L of distilled water and heated to 40 °C. The solution was then sterilized in an autoclave at 120 °C and 0.5 atm for 30 min, after which it was cooled and stored in a refrigerator at 8 °C. Fungi were cultivated using potato dextrose agar (PDA) and Czapek medium (both obtained from Titan Biotech Ltd., Delhi, India). The content of NF in the samples was determined using the standard surface seeding technique [28]. Soil samples weighing 0.5 to 1 g were placed in the center of a layer of WA in Petri dishes using a scalpel. The primary cultures were maintained at room temperature for the first seven days. After this period, Meloidogyne incognita Kofoid and White nematodes were added, and the cultures were incubated in a thermostat at 23–25 °C for three months. The presence and growth of NF in the samples were assessed under a microscope. The obtained NF strains were identified at the species level based on morphological features, following the methods described by Cook, Godfrey, and Duddington [29,30].

### 2.3. Test Object—Phytoparasitic Nematodes

**Figure 2:**
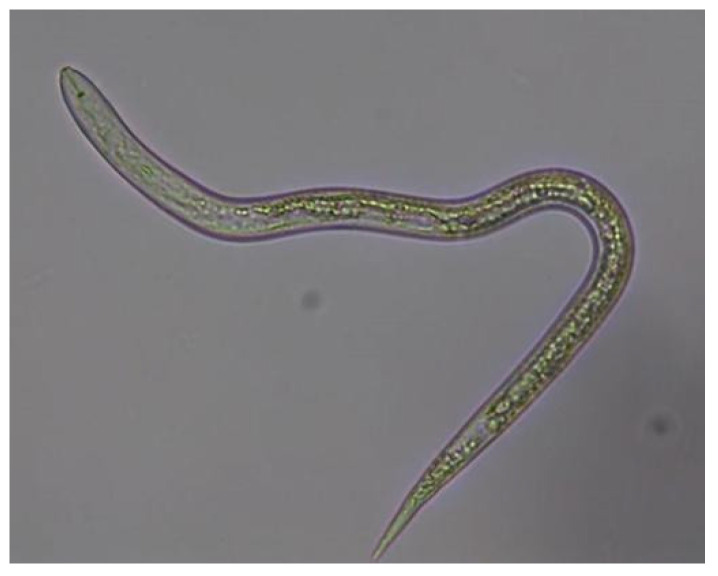
Adult nematode—*M. incognita*.

Samples of the nematode *M. incognita* were obtained from the collection of the Estación Experimental del Zaidín (Figure 2).

The nematode culture was prepared following the method outlined by Chronis et al. [31]. Infected tomato plants were sourced from plots that displayed signs of disease. The infected roots were cut into 1–2 cm fragments, and eggs were released by blending these roots in a 1% sodium hypochlorite solution. After incubating for 10 min, distilled water (40 volumes) was added to the egg suspension, which was then sieved through 100 μm and 25 μm mesh sizes. The eggs were collected in a glass beaker and left in sterilized distilled water for three days to facilitate the hatching process.

### 2.4. Attractive Activity of NF

To assess the attractive activity of the isolated NF, we utilized the following materials: (1) sterile Petri dishes, (2) sterile water, (3) an agar disk containing a fungal strain that measured 1.5 cm in diameter, and (4) a nematode culture diluted in 5 mL of water [32]. In the experiments, the agar disk with the fungal strain was placed face down in the center of a sterile Petri dish, which was then filled with distilled water up to the level of the disk’s surface. Next, 0.1 mL of the nematode culture, containing 90 specimens, was added to each Petri dish.

For the negative control, nematode cultures were introduced into Petri dishes containing distilled water (Control 1) and distilled water + PDA (Control 2) without NF.

The results regarding attractiveness were evaluated under a stereo microscope at a magnification of 7× after 2, 4, 10, and 24 h by counting the number of nematodes surrounding the disk.

### 2.5. Nematophagous Activity of NF

To assess the nematophagous activity of the isolated fungal strains, we utilized the method described by T.M. Teplyakova [33]. A 3 mm diameter disk of the fungal culture was cut out and placed in a suspension containing 90 specimens of *M. incognita* nematodes. After 24, 48, and 72 h, we counted the number of live nematodes in the Petri dishes, specifically in areas without the fungus.

Following these counts, the nematophagous efficiency (Ne) was calculated using the following formula:

$$Ne=1-\frac{K_{1}\times K_{c}}{K_{0}\times K_{2}}\times 100,$$

where

N_e_—nematophagous efficiency in %;

K_0_—the number of live nematodes before adding the NF;

K_1_—number of living nematodes after 24 h;

K_c_—the number of living nematodes in control before the introduction of the NF;

K_2_—the number of living nematodes in the control after the introduction of the NF after 24 h.

### 2.6. Statistical Analysis

Statistical analysis of nematophagous activity was performed using two-way ANOVA (IBM SPSS ver.24). The correlation between NF attractivity and trapping activity was assessed with Pearson correlation.

### 2.7. Identification of NF Strains

The strains were identified by determining the direct nucleotide sequence of the nuclear ribosomal internal transcribed spacer (ITS) region, followed by determination of nucleotide identity with sequences deposited in the international GeneBank database.

### 2.8. NF DNA Extraction

NF cultures aged two to four days were transferred from Petri dishes into 2 mL plastic test tubes and then frozen at −20 °C overnight. After freezing, the samples were allowed to thaw at room temperature. Next, 500 μL of lysis buffer (Titan Biotech Ltd., Delhi, India) and 5 μL of proteinase (Titan Biotech Ltd., Delhi, India) were added to the samples. The mixture was then homogenized and incubated overnight at 65 °C. After incubation, the samples were cooled to room temperature.

Subsequently, 750 μL of a chloroform and isoamyl alcohol mixture (24:1) were added. The samples were shaken and then centrifuged at 10,000 rpm for 20 min at 4 °C. After centrifugation, the aqueous phase was collected, and an equal volume of a phenol, chloroform, and isoamyl alcohol mixture (24:25:1) was added. The samples were centrifuged again under the same conditions, and the aqueous phase was carefully collected into sterile test tubes.

To precipitate the DNA, 0.6 volumes of isopropanol were added, followed by centrifugation at 10,000 rpm for 20 min at room temperature. The resulting DNA precipitate was washed twice with 70% ethanol. The purified DNA sample was then dissolved in 100 μL of 1× TE buffer and stored at −20 °C. Finally, DNA concentration was measured using a NanoDrop spectrophotometer (Thermo Fisher Scientific Inc., Waltham, MA, USA) at a wavelength of 260 nm.

### 2.9. PCR Analysis and Amplification of the ITS Region

The PCR reaction was conducted using universal primers [34] ITS4-5′—GGAAGTAAAAGTCGTAACAAGG—3′ and ITS5-5′—TCCTCCGTTATTGATGC—3′. The total volume of the reaction was 25 μL, utilizing the 2× BioMaster HS-Taq PCR kit (Biolabmix LLC, Novosibirsk, Russia) according to the manufacturer’s instructions. The PCR mixture included 150 ng of DNA and 15 pmol of each primer.

The PCR amplification program consisted of a long denaturation step at 95 °C for 5 min, followed by 30 cycles of the following steps: denaturation at 95 °C for 20 s, annealing at 52 °C for 30 s, and extension at 68 °C for 3 min. A final elongation step was performed at 68 °C for 7 min. The PCR program was executed using a BioRad amplifier (BioRad Laboratories Inc., Hercules, CA, USA).

### 2.10. Determination of the Nucleotide Sequence

Purification of PCR products from unbound primers was carried out using the enzymatic method [35]. The enzymes utilized were Exonuclease I and FastAP thermosensitive alkaline phosphatase (both obtained from Thermo Fisher Scientific Inc. in Waltham, MA, USA).

The sequencing reaction was conducted using the BigDye™ Terminator v3.1 Cycle Sequencing Kit (Thermo Fisher Scientific Inc., Waltham, MA, USA), following the manufacturer’s instructions. Subsequently, the fragments were separated using the 3730xl DNA Analyzer (Thermo Fisher Scientific Inc., Waltham, MA, USA).

### 2.11. Analysis of Nucleotide Sequences

For the analysis of nucleotide sequences, the nucleotide sequences of the ITS region from the identified strain were compiled into a common sequence using SeqMan software, Lasergene 6.0 (DNASTAR Inc. in Madison, WI, USA). Terminal fragments were removed, including the nucleotide sequences of the primers and fragments with low-quality scores. This process resulted in a nucleotide sequence longer than 650 bp. Finally, the sequences were identified in GeneBank using the BLAST algorithm [36].

## 3. Results

### 3.1. Isolation of NF

**Figure 3:**
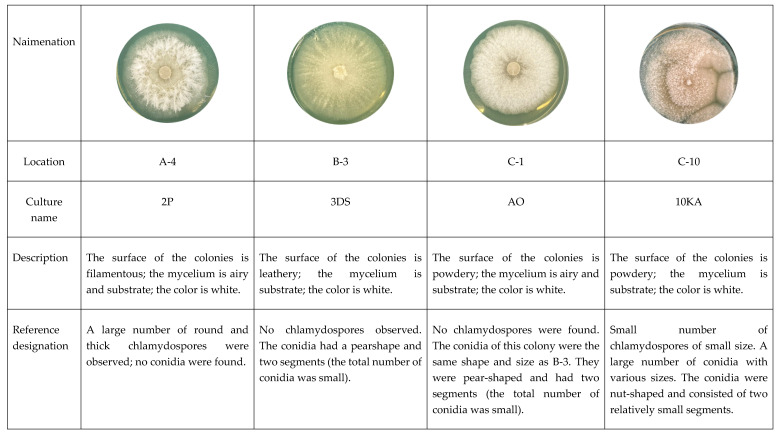
Description and reference designation of isolated cultures of NF.

Figure 3 displays isolated primary colonies of predatory fungi grown on PDA medium. Strains that synthesize attractive and toxic substances more actively show a faster trapping effect in the presence of nematodes, whether in an aqueous suspension or on the surface of an aqueous agar solution. During the observations, several developmental stages were recorded: germination of conidia, mycelial growth and trap formation, and the development of conidiophores, conidia, and chlamydospores. Typically, chlamydospores are the first to form among all nematodes.

**Figure 4:**
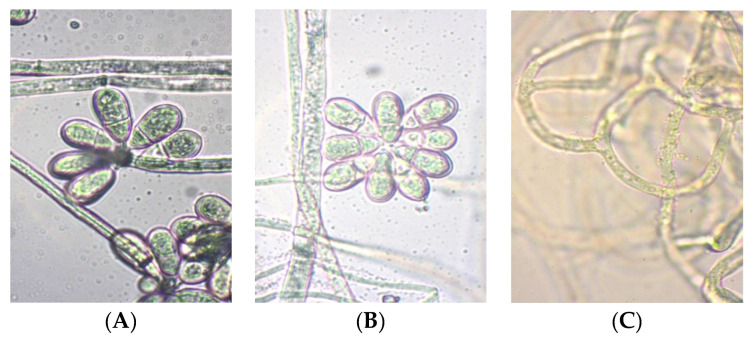
Different forms of conidia and adhesive loops of the nematophagous fungus strain C-1 (AO). (**A**) Head-shaped conidia; (**B**) separate clusters of conidia; (**C**) adhesive loops.

The cultural and morphological characteristics of isolated preparations of nematophage fungi are illustrated using strain C-1 (AO) (Figure 4), grown on PDA. The colonies in Petri dishes developed a dense, slightly raised white mycelium with smooth edges, resembling cotton wool. At three days old, the colony measures 30 mm; at five days, it measures 55 mm, and at ten days, it reaches 90 mm. Sporulation occurs with conidia formation, while the colony’s reverse side exhibits colors ranging from light sand to light brown. As the colony ages, its color gradually shifts to a slightly sandy hue.

The mycelial hyphae are smooth, measuring 4–8 μm in diameter, and conidia develop on simple, straight conidiophores that range from 300 to 500 μm in length and vary from 7–12 μm at the base to 4–6 μm at the distal end. The conidia are two-celled and elongated-pear-shaped, with the distal cell measuring 25–27 μm by 10–14 μm, nearly twice the size of the proximal cell. Conidia are gathered in two distinct forms at the tops of the conidiophores: in heads of 8–12 conidia (Figure 4A) and in separate clusters of 5–10 conidia (Figure 4B).

Based on the morphological characteristics and the identification keys provided by Cook, Godfrey, and Duddington, the strain is classified under the genus *Orbilia*. In soil and culture, the strain produces thick-walled chlamydospores on nematodes, which are oval or round and measure between 25 and 35 μm. In the presence of nematodes, the hyphae develop rounded loops that interweave to form a net-like structure. The average internal size of these capturing structures ranges from 25 to 30 μm, although some rounded rings with diameters of up to 40 μm have also been observed (Figure 4C).

### 3.2. Assessment of the Growth Rate, Attractive and Trapping Activity of NF

**Figure 5:**
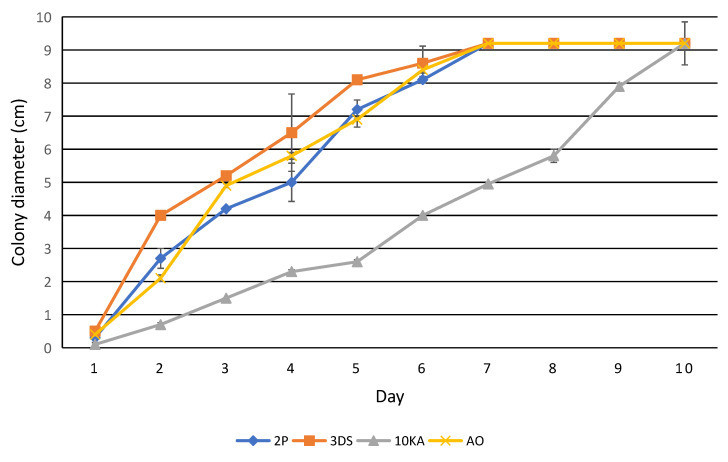
The growth rate of NF on PDA.

Samples 2P, 3Ds, and AO exhibited similar growth dynamics during the first week of cultivation. By the 7th day, the colonies had spread to cover the entire plate, with an average colony diameter of 9.2 to 9.4 cm. In contrast, the growth of strain 10KA was slower than that of the other samples. However, by the end of the 10th day, it covered the entire plate, achieving a colony diameter of 9.2 to 9.4 cm. These growth indicators are consistent with normal growth patterns of NF under the experimental conditions (see Figure 5).

**Figure 6:**
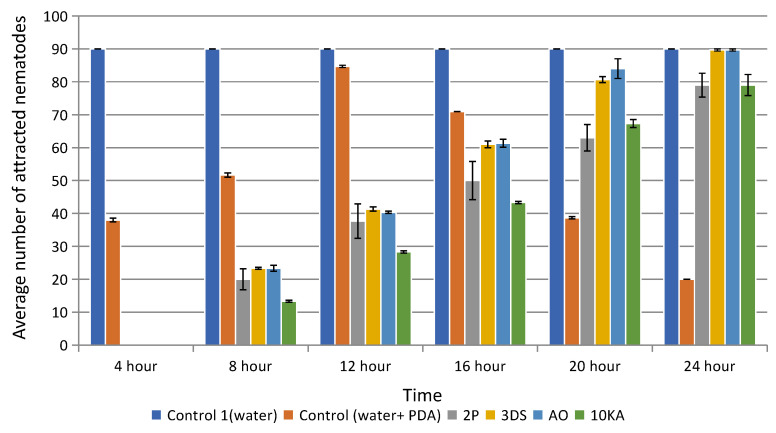
Attractive activity of isolated strains of NF. Error bars indicate standard deviation. Control 1 and 2 do not contain fungi. Statistically significant difference represent between strains 3DS and AO, and 2P and 10KA (*p* ≤ 0.05).

Based on the results, all the fungi strains tested exhibited high levels of attractive activity (Figure 6). By the third day of cultivation, nearly all the nematodes had gathered around the agar disk, confirming the viability and functional activity of the utilized NF strains. The experiments also showed the dynamics of changes in the attractive activity of various strains of NF depending on the incubation time.

**Figure 7:**
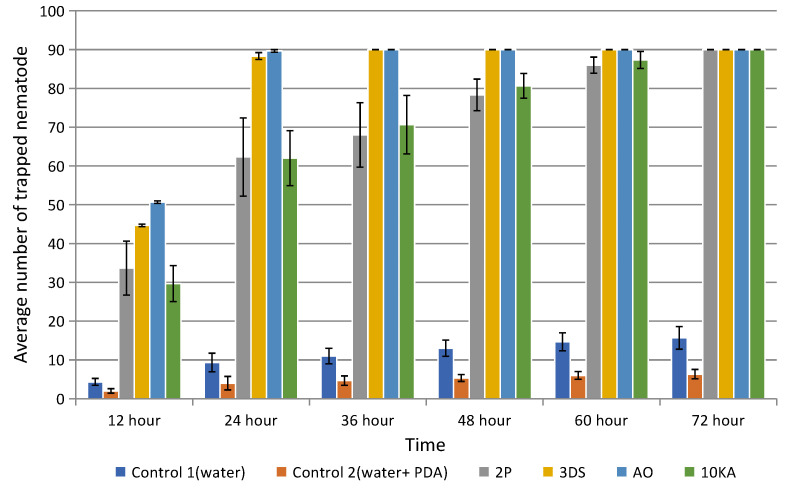
Nematophagous activity of isolated fungal strains. Error bars indicate standard deviation. Statistically significant difference represent between strains 3DS and AO, and 2P and 10KA (*p* ≤ 0.05).

NF used in the experiments shows high efficiency in catching nematodes, starting from 12 h and stable activity (80–90 nematodes) by 72 h (Figure 7). Strains 3DS and AO showed the highest trapping activity (*p* ≤ 0.05), capturing all available nematodes within 24–36 botrys superbah. Compared to them, strains 2P and 10KA showed lower trapping activity, capturing all available nematodes only within 60–72 h. The spread of data (reflected by standard deviations) in the experiments is insignificant, which indicates a high level of reproducibility of the results. 

The correlation between attractiveness and predation increased with the duration of the interaction between nematodes and fungi. Thus, a strong negative correlation was observed after 12 h of co-cultivation, but a positive correlation was found after 24 h of co-cultivation.

### 3.3. Molecular Phylogenetic Identification of NF

**Figure 8:**
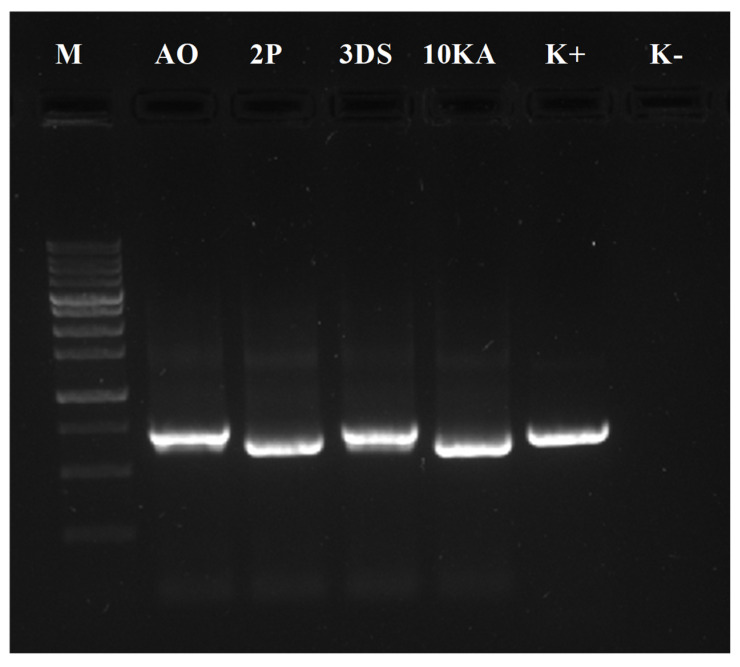
Electrophoresis of PCR products of ITS region amplification of DNA samples. Designations: (M) molecular weight marker (Fermentas) (250–10,000 bp, from 250 to 1000 step 250 bp), AO-10KA—samples, (K+)—positive control (K−)—negative control.

Four of the most active strains were selected for molecular phylogenetic identification with the following designations: AO, 2P, 3DS, and 10KA. The analysis of PCR amplification of the 16S rRNA gene fragment revealed that fragments approximately 700 bp in size were amplified from the samples (Figure 8).

Identification of strains of NF based on primary nucleotide sequence analysis was performed in the GenBank database using the BLAST program. As a result, isolated strains of NF inhabiting the soils of agricultural lands in Southern Kazakhstan were identified (Table 2).

The results of strains were conducted using nucleotide sequence analysis of the nuclear ribosomal ITS region, which served as a molecular biological characteristic of the NF strains. Sequencing for these samples was performed multiple times.

**Figure 9:**
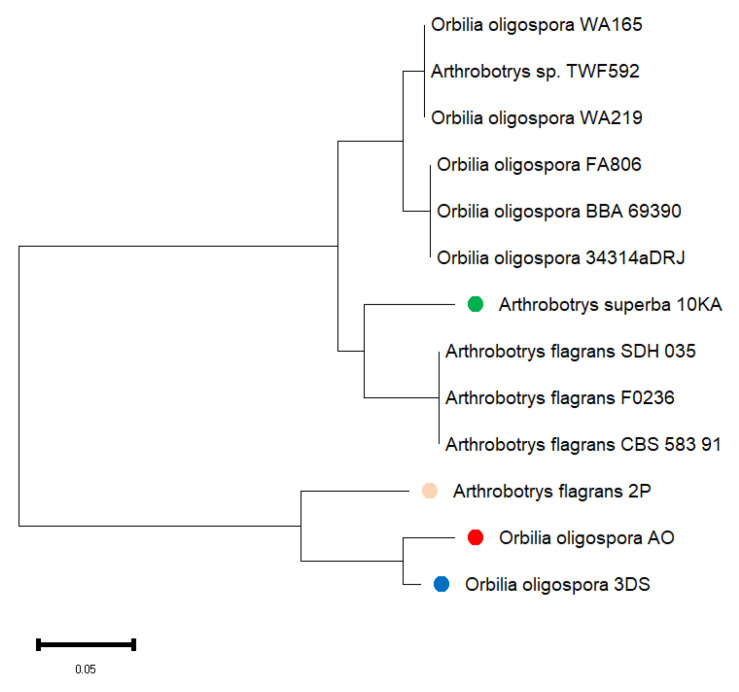
Phylogenetic tree of the ITS region of isolated NF. Colored circles indicate the isolated strains: Green: *Arthrobotrys superba* (PP740690); pink: *Arthrobotrys flagrans* (PP748482); red: *Orbilia oligospora* (PP740634); blue: *Orbilia oligospora* (PP740516). Phylogenetic tree constructed using MEGA11 (Ver. 11.0.13).

The nucleotide sequence of the nuclear ribosomal ITS region was utilized to reconstruct the phylogenetic tree, depicting the relationships among the isolated fungal strains (Figure 9).

Analysis of the obtained results showed that all four isolated strains of nematophagous fungi (identity from 98.8% to 100%) belong to the genus Arthrobotrys (former name Orbilia) and almost completely correspond to the species indicated in the figure. The phylogenetic relationship of the isolated new strains of nematophagous fungi of South Kazakhstan with *Arthrobotrys* sp. does not raise doubts. No new species of nematophagous fungi were found on cultivated lands.

## 4. Discussion

The study successfully isolated and identified NF from the soils of agricultural lands in Southern Kazakhstan, highlighting their potential for biological control of plant-parasitic nematodes. These findings align with global efforts to develop sustainable alternatives to chemical nematicides, which are known for their toxicity, environmental persistence, and reduced efficacy due to the development of resistance. For instance, a French company has released Royal 300^®^, which is based on the non-mammalian fungus *A. robusta* var. antipolis to combat the *Ditylenchus myceliophagus* nematode [37]. Another product, Royal 350^®^, utilizes *Arthrobotrys irregularis* to biocontrol *Meloidogyne* nematodes in tomatoes [38]. Identifying *O. oligospora*, *D. flagrans*, and *A. superba* as effective bionematicides underscores the adaptability and efficiency of local fungal strains in combating nematodes in the unique soil conditions of Southern Kazakhstan. This approach is consistent with previous studies demonstrating the advantages of using locally adapted strains for pest control, as these strains exhibit higher survival and efficacy rates under regional environmental conditions [39].

The morphological observations in this study, which include the formation of adhesive loops and trapping structures, are comparable to research conducted by [30]. They emphasized the importance of trap diversity in NF for effective nematode capture. Moreover, molecular identification through ITS sequencing has confirmed the identity of the fungal strains, showing a close correlation with sequence data from GenBank (98.8–100% similarity). This finding supports the reliability of molecular tools for precise taxonomic classification, as suggested by Zhang et al. [39]. The molecular phylogenetic identification revealed that the AO and 3DS strains are *O. oligospora*, the 2P strain is *D. flagrans*, and the 10KA strain is *A. superba*. The ITS region sequences for these four strains have been deposited in GenBank under PP740634, PP748482, PP740516, and PP740690.

The effectiveness of *O. oligospora* observed in this study aligns with findings from Yao et al. [40], who reported that the volatile compounds produced by *O. oligospora* attract nematodes, thereby increasing its predatory efficiency. Similarly, our findings regarding the rapid trap formation and high nematophagous efficiency of *D. flagrans* align with those of Rahman et al. [41], who identified this fungus as a promising bionematicide due to its ability to form robust chlamydospores, ensuring survival in diverse environmental conditions.

In contrast, the performance of *A. superba* in our experiments was relatively moderate, capturing nematodes over a longer time frame (60–72 h). This differs from reports by Li et al. [42], which highlighted the superior predation efficiency of *A. superba* in soils with higher organic matter content. These discrepancies may stem from differences in soil composition, as Southern Kazakhstan soils are characterized by lower humus content in many regions.

However, the reasons behind the highly attractive and nematophagous activities of the 3DS and A.O. strains, compared to the other tested strains, are not fully understood and require further investigation. Identifying these factors in future studies is crucial for understanding the traits that need to be developed in NF to enhance the productivity of bionematicides based on them.

## 5. Conclusions

The results of the isolation, morphological characteristics, and molecular phylogenetic analysis of NF living in the soils of agricultural lands in Southern Kazakhstan are consistent with similar data presented in the world scientific literature on the study of the relationship between NF and nematodes.

For the first time in Kazakhstan, data on the presence and species composition of NF capable of carrying out biocontrol of phytoparasitic nematodes during the cultivation of crops have been presented. Identified the following types of NF: *O. oligospora*, (Kyzylorda region), *A. flagrans* (Zhambyl region), *O. oligospora* (Almaty region), *A. superba* (Almaty region).

These strains will be used to prepare a bionematicide, which will be tested in a greenhouse with the addition of certain doses of vermicompost (biohumus) and then in experimental plots in places of detection.

## Figures and Tables

**Table 1:** Soil sampling points in the territory of Southern Kazakhstan.

Location/Habitats	Site	GPS Coordinates
Kyzylorda region/Fruit garden	A-1, A-2, A-3	44°49′15.5″ N 65°30′27.9″ E
Kyzylorda region/Vegetable garden	A-4, A-5, A-6	44°49′15.5″ N 65°30′27.9″ E
Chu district, Orazaly village, Zhambyl region/Potato field	B-1, B-2, B-3	43°27′4436 N 74°15′4509 E
Chu district, Orazaly village, Zhambyl region/Barley grown in soil	B-4, B-5, B-6	43°27′4436 N 74°15′4509 E
Chu district, Orazaly village, Zhambyl region/Cucumbers grown in soil	B-7, B-8, B-9	43°27′4436 N 74°15′4509 E
Chu district, Orazaly village, Zhambyl region/Garlic grown in soil	B-10, B-11, B-12	43°27′4436 N 74°15′4509 E
Kazakh Research Institute of Potato and Vegetable Growing, Almaty region/Potato field	C-1, C-2, C-3	43°15′8993 N 76°44′878 0E
Kazakh Research Institute of Potato and Vegetable Growing, Almaty region/Alfalfa grown in soil	C-4, C-5, C-6	43°15′8993 N 76°44′8780 E
Kazakh Research Institute of Potato and Vegetable Growing, Almaty region/Cabbage grown in soil	C-7, C-8, C-9	43°15′8993 N 76°44′8780 E
Kazakh Research Institute of Potato and Vegetable Growing, Almaty region/Corn	C-10, C-11, C-12	43°15′8993 N 76°44′8780 E

**Table 2:** Results of ITS sequence identification.

Name Cultures	Result Identification in BLAST		
	Accession GeneBank	Strain Name	% Ident
AO (PP740634)	OQ248188.1	*Orbilia oligospora* strain FA806	98.98%
	MF782733.1	*Orbilia oligospora* strain 34314aDRJ	98.81%
	KY463695.1	*Orbilia oligospora* voucher BBA 69390	98.81%
2P (PP748482)	MK156709.1	*Duddingtonia flagrans* isolate F0236	100.00%
	KT215213.1	*Arthrobotrys flagrans* strain CBS 583 91	100.00%
	KP257593.1	*Duddingtonia flagrans* isolate SDH 035	100.00%
3DS (PP740516)	MN014034.1	*Arthrobotrys* sp. isolate TWF592	100.00%
	OQ244205.1	*Orbilia oligospora* strain WA219	100.00%
	OQ244200.1	*Orbilia oligospora* strain WA165	100.00%
10KA (PP740690)	KT215210.1	*Arthrobotrys superba* strain CBS 109 52	99.83%

## Data Availability

The data presented in this study are available on request from the corresponding author. The data are not publicly available due to being located in laboratory journals.
